# The inhibitory effect of Isoliquiritigenin on the proliferation of human arterial smooth muscle cell

**DOI:** 10.1186/s40360-017-0165-2

**Published:** 2017-07-17

**Authors:** Tianbao Chen, Shaoxiong Deng, Rong Lin

**Affiliations:** 1 Department of Cardiology, First Hospital of Quanzhou Affiliated to Fujian Medical University, NO. 205, East Street, Licheng District, Quanzhou, Fujian China; 2 Quanzhou Medical College, NO. 2, Anji Street, Luojiang District, Quanzhou, Fujian China

**Keywords:** Isoliquiritigenin, HASMCs, Proliferation, Oxidative stress, PI3K signaling

## Abstract

**Background:**

Isoliquiritigenin (ISL) has various biological activities including as antioxidant and an inhibitor of PI3K/AKT signaling pathway. However, both oxidative stress and activated PI3K/AKT signaling contribute to the aberrant proliferation of vascular smooth muscle cells (VSMCs). This study is aimed to explore the effect of ISL on the proliferation of human arterial smooth muscle cells (HASMCs) and to investigate the underlying mechanisms.

**Methods:**

BrdU incorporation, cell cycle and reactive oxygen species (ROS) in normal or ISL treated HASMCs were analyzed by flow cytometry. Cell viablity was measured by CCK-8. Protein expression levels were examined by Western blot, and superoxide dismutase (SOD) activity was detected by using commercial kit.

**Results:**

We observed that ISL could inhibit the proliferation of HASMCs in a dose and time dependent manner. Cell cycle of ISL treated HASMCs arrested mainly in G1/S phase and accompanied with elevated expression of p27 and decreased expression of CyclinD1 and CyclinE. In addition, ISL could down-regulated the expression of p-PI3K and p-AKT, alleviated oxidative stress and enhanced the SOD activity in HASMCs. Furthermore, H_2_O_2_ treatment partly improved cell viability and up regulated p-PI3K and p-AKT in HASMCs.

**Conclusions:**

Therefore, we concluded that ISL inhibited the proliferation of HASMCs via attenuating oxidative stress and suppressing PI3K/AKT signaling pathway. The inhibitory effect of ISL on PI3K/AKT signaling pathway, at least partly, was mediated by ROS.

## Background

The aberrant proliferation of VSMCs is thought to be a key event in plaque formation of atherosclerosis [1]. Both in vitro and in vivo studies indicate that inhibiting VSMCs proliferation could be a strategy for delaying atherosclerosis progression [2–4]. The underlying mechanisms and molecules involved in cell-cycle regulation of VSMCs had been studied a lot. Among which, oxidative stress is generally acknowledged as one of primary contributors. Multiple studies had demonstrated that eliminating of the excessive ROS could decrease the proliferation of VSMCs significantly [5–7].

Isoliquiritigenin (ISL) from licorice compounds showed various biological activities including antioxidant and anti-inflammatory properties [8, 9]. Previous study has revealed ISL could significantly inhibit cytokine-induced endothelial cell adhesion molecule expression [10]. Further study has demonstrated that the inhibitory effect of ISL on cell adhesion molecule expression, partly depended on its function in blocking ROS generation [11]. Besides antioxidant and anti-inflammatory properties, ISL is regarded as a nature inhibitor of PI3K/AKT signaling pathway in breast cancer [12]. And, PI3K/AKT signaling pathway is also necessary for VSMCs proliferation [13–15]. Taken together, we propose that ISL may effectively suppress the proliferation of VSMCs through inhibiting oxidative stress and molecules in PI3K/AKT signaling. It has been reported that ROS could modulate PI3K/AKT signaling pathway via several manners such as deactivating PTEN or protein phosphatase 2A [16–18]. Therefore, we will investigate whether the inhibitory effect of ISL on PI3K/AKT signaling pathway is mediated by ROS.

In present study, we report that: 1) ISL inhibits the proliferation of HASMs in a dose and time dependent manner. 2) Inhibitory effect of ISL on HASMCs proliferation at least partly depends on attenuating oxidative stress and suppressing PI3K/AKT signal pathway. 3) The modulation of PI3K/AKT pathway by ISL is partly mediated by ROS. Together, these findings will provide theory and experimental basis for ISL clinical use in treatment of atherosclerosis.

## Results

### Effect of Isoliquiritigenin on human aortic smooth muscle cell viability

**Fig. 1:**
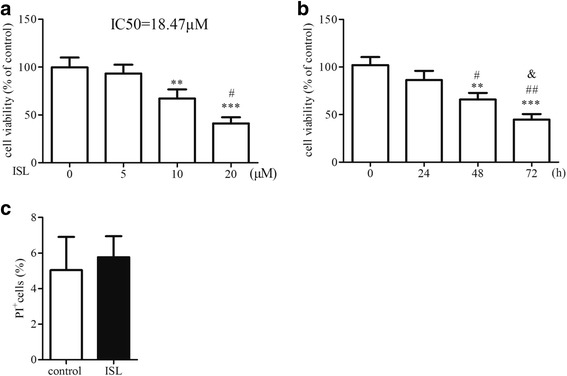
Effect of Isoliquiritigenin on human aortic smooth muscle cell expansion. **a** After incubation with various concentrations of ISL for 48 h, HASMCs cell viability was examined by CCK-8 assay. **b** HASMCs were treated with ISL (10 μM) for different time points and the cell viability was detected by cck8 assay. **c** HASMCs were treated with ISL (10 μM) for 48 h and cell death was determined by PI staining. All values represented as mean ± SD, *n* = 5, **: *P* < 0.01; ***: *P* < 0.001 compared with untreated HASMCs. #: *P* < 0.05; ##: *P* < 0.01, for (**a**) compared with 10 μM ISL treated HASMCs; for (**b**) compared with ISL treated HASMCs for 24 h. &: *P* < 0.05 compared with ISL treated HASMCs for 48 h

We first examined effect of different dose of ISL on HASMCs cell viability. Results showed that ISL inhibited cell viability of HASMCs in a dose dependent manner and the IC50 of ISL to HASMCs was 18.47 μM (Fig. 1a). Subsequently, 10 μM ISL was used to treat HASMCs at different time points and we observed that ISL also inhibited cell viability of HASMCs in a time dependent manner (Fig. 1b).

To identify whether decreased HASMCs viability induced by ISL was associated with increased cell death, we performed PI staining. Result showed that ratio of PI positive cells in ISL treated HASMCs was a little higher than that in ISL untreated cells. This result demonstrated that ISL didn’t induce cell death.

### Effect of Isoliquiritigenin on cell cycle progression of HASMCs

**Fig. 2:**
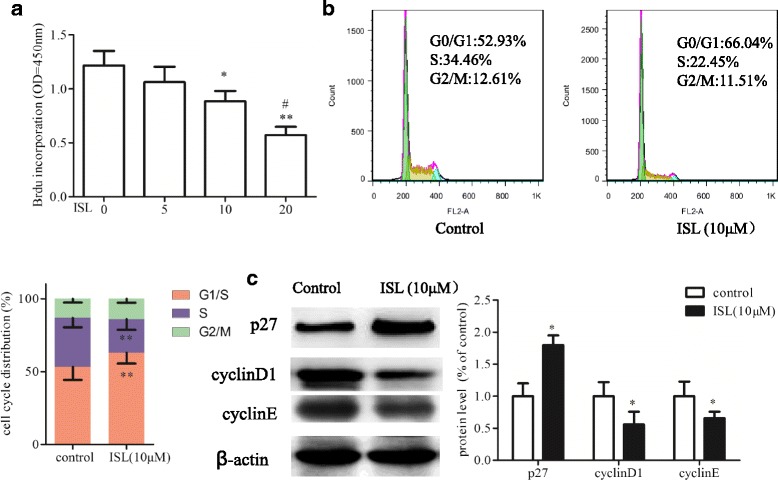
Effect of Isoliquiritigenin on cell cycle progression of HASMCs. **a** HASMCs were treated with various concentration of ISL for 48 h, and incorporated with BrdU. BrdU content in cells was detected by enzyme-linked immunosorbent assay. After incubation with or without ISL (10 μM) for 48 h, **b** HASMCs were stained with PI for cell cycle analysis, **c** cell extracts were analyzed by Western-blot for detecting expressions of p27, CyclinD1 and CyclinE. Protein levels were calculated as a ratio relative to β-actin and expressed relative to untreated HASMCs. All values represented as mean ± SD, *n* = 5, *: *P* < 0.05; **: *P* < 0.01 compared with untreated HASMCs. #: *P* < 0.05 compared with 10 μM ISL treated HASMCs

To explore effect of ISL on cell cycle progression, we first investigated DNA synthesis in ISL treated or untreated HASMCs by BrdU incorporation. Results showed that BrdU inhibited HASMCs DNA synthesis in a dose dependent manner. (Fig. 2a). Cell cycle analysis revealed that ISL significantly increased the ratio of G1/S cells and reduced the cells in S phase (Fig. 2b). These results demonstrated that ISL could significantly block the progression of cell cycle.

To explore whether ISL induced cell cycle blockage attributed to changes of cell cycle regulatory molecules, we checked the expressions of p27, CyclinD1 and CyclinE in ISL treated or untreated HASMCs. Results showed that the expression of p27 was up-regulated in ISL treated HASMCs compared to untreated cells. In contrast, the expressions of CyclinD1 and CyclinE were down-regulated in ISL treated cells (Fig. 2c). These results demonstrated that ISL induced cell cycle arrest via modulating cell cycle regulatory molecules.

### Effect of Isoliquiritigenin on ROS production in HASMCs

**Fig. 3:**
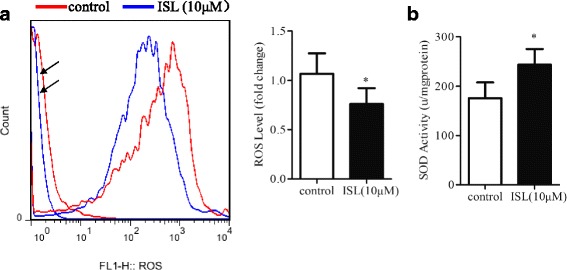
Effect of Isoliquiritigenin on ROS production. **a** Flow cytometry analysis of reactive oxygen species in ISL (10 μM) treated or untreated HASMCs for 48 h. DCFDA was used for intracellular ROS labeling. The ROS levels were calculated as a ratio of relative to untreated HASMCs. **b** Total SOD enzyme activity in cells were determined by SOD activity test. All values represented as mean ± SD, *n* = 5, *: *P* < 0.05 compared with untreated HASMCs. *Arrows* indicated autofluorescence

Considered that ROS is required for VSMC proliferation and ISL has a property of antioxidant. We examined ROS level by flow cytometry in ISL treated or untreated HASMCs. Results showed that ROS level was reduced significantly by ISL treatment (Fig. 3a). Furthermore, SOD activity was obvious higher in ISL treated HASMCs than that of untreated cells (Fig. 3b).

### Effect of Isoliquiritigenin on signaling pathway related to PI3K/AKT

**Fig. 4:**
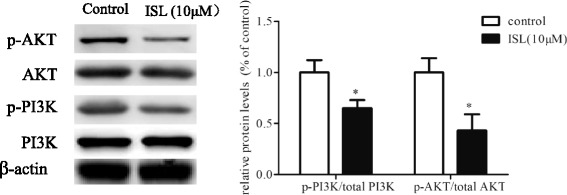
Effect of Isoliquiritigenin on signaling pathway related to PI3K/AKT. Western blot analysis of cell extracts from ISL (10 μM) treated or untreated HASMCs for 48 h for detecting expressions of PI3K, p-PI3K, AKT, p-AKT. Phosphorylated protein levels were calculated as a ratio relative to total protein and expressed relative to untreated HASMCs. All values represented as mean ± SD, *n* = 5, *: *P* < 0.05; **: *P* < 0.01 compared with untreated HASMCs

ISL is reported as an inhibitor for PI3K-AKT signaling pathway [12]. Therefore, we wondered that if there are any molecular alternations of PI3K-AKT signaling pathway between control and ISL treated HASMCs. Western blot analysis revealed that although total expression of PI3K or AKT were not affected by ISL treatment, both the expressions of p-PI3K and p-AKT were down-regulated in ISL treated HASMCs (Fig. 4).

### H_2_O_2_ abolished the inhibitory effect of Isoliquiritigenin on HASMCs proliferation

**Fig. 5:**
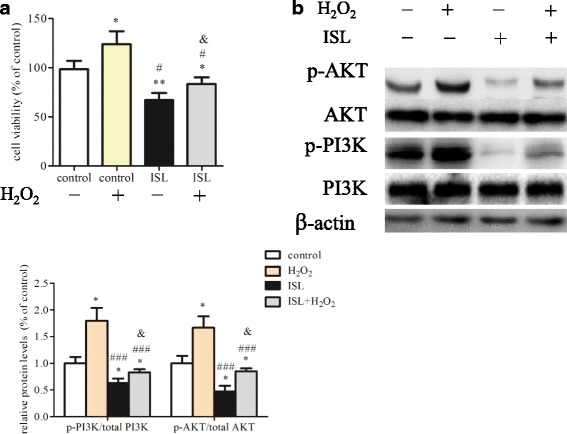
H_2_O_2_ abolished effect of Isoliquiritigenin on HASMCs proliferation. After incubation with or without ISL (10 μM) in the absence or presence of H_2_O_2_ (100 μM) for 48 h, **a** HASMCs viability was examined by cck-8 assay. **b** Western blot analysis of cell extracts for detection expressions of PI3K, p-PI3K, AKT, p-AKT. Phosphorylated protein levels were calculated as a ratio relative to total protein and expressed relative to untreated HASMCs. All values represented as mean ± SD, *n* = 5, *: *P* < 0.05; **: *P* < 0.01 compared with untreated HASMCs. #: *P* < 0.05; ###: *P* < 0.05 compared with 100 μM H_2_O_2_ treated HASMCs. &: *P* < 0.05 compared with ISL treated HASMCs

To explore whether H_2_O_2_ could restore the proliferation of HASMCs, we treated cells with H_2_O_2_, ISL or ISL combined with H_2_O_2_. CCK8 assay revealed that H_2_O_2_ abolished the inhibitory effect of_._ ISL on HASMCs proliferation (Fig. 5a). Western blot analysis revealed that the decreased expressions of p-PI3K and p-AKT in ISL treated HASMCs were partly corrected by H_2_O_2_ administration (Fig. 5b).

## Discussion

ISL, one of the major active components in licorice, possesses multiple biological activities including anti-oxidant, anti-inflammation and anti-tumor [8, 9, 19]. Several studies indicated the protective effects of ISL on cardiac and vessels, such as enhancing the cardiac and aortic muscle contractility, dampening cardiac injury caused by ischemia-reperfusion and down regulating cytokine induced cell adhesion molecules expression in endothelial cell [11, 19–21]. However, the effect of ISL on VSMCs proliferation remains to be investigated. In the present study, we found that ISL inhibited the HASMCs proliferation via blocking cell cycle progression. These results are similar to the inhibitory effect of ISL on proliferation of endothelia cells [22].

The cell cycle is tightly regulated by cell cycle regulatory molecules such as phosphorylated retinoblastoma protein (pRB), cyclins, cyclin dependent kinase (CDK) and CDK inhibitors (CDKI) [23]. The G1/S transition is mainly controlled by CyclinD-CDK4 and CyclinE-CDK2 complexes [24]. And, the interaction between CDK and cyclins could be inactivated by CDKI, such as p21 or p27 [25]. Here, we found that ISL could induce the cell cycle arrest of HASMCs in G1/S phase. Further exploration demonstrated that ISL down regulated the expressions of cyclinD1 and cyclinE in HASMCs whereas up regulated p27 expression.

It is well known that oxidative stress-induced proliferation of VSMCs plays a critical role in pathogenesis of atherosclerosis [26–28], and a mount of molecules were demonstrated to inhibit HASMCs proliferation mainly via attenuating oxidative stress [5, 7, 29–32]. Here, we found that ISL could effectively lowered ROS level in HASMCs and this may attribute to the increased SOD activity. Furthermore, H_2_O_2_ treatment recovered the proliferative ability of ISL-treated HASMCs. However, the exact mechanisms of ISL on oxidative stress regulation remain to be investigated.

PI3K/AKT signaling pathway is associated with cell proliferation and survive [33, 34]. Recent studies have identified that ISL is a kind of nature inhibitors of PI3K/AKT signaling in breast cancer [12]. We found that ISL administration could down regulated the expression of molecules in PI3K/AKT signaling pathway, including p-PI3K and p-AKT. Previous studies reveal that ROS could modulate the PI3K/AKT pathway through inactivating PTEN [17]. In consistent with this, we found that H_2_O_2_ addition partly rescued the expressions of both p-PI3K and p-AKT in ISL treated HASMCs.

It has been noted that ISL also exhibit significant estrogenic property. Estrogen receptors such as ERα and ERβ are expressed in VSMC [35]. Several studies have demonstrated that estrogen can inhibit the proliferation of VSMC via binding estrogen receptors, thereby protecting cardiovascular system from cardiovascular events [36, 37]. Estrogen signaling pathway can activate both PI3K/AKT pathway and antioxidant system in cells [38, 39]. However, whether the inhibitory effect of ISL on HASMCs proliferation depend on its estrogenic activity remains to be investigated.

In conclusion, we demonstrated that ISL could inhibit the proliferation of HASMCs through alleviating ROS and down-regulating molecules in PI3K/AKT signaling pathway. Furthermore, the inhibitory effect of ISL on PI3K/AKT signaling pathway, at least partly, depended on its function on ROS scavenging.

## Conclusion

This is the first paper toward investigating the role of ISL in HASMCs proliferation. Our results showed that ISL specifically inhibited the proliferation of HASMCs in a dose and time dependent manner. Further study demonstrated that ISL arrested cells cycle in G1/S phase via up-regulating p27 and down-regulating CyclinD1 and CyclinE. These results are consistent with the previous report which investigates the effect of ISL on proliferation of endothelia. It is generally accepted that both ROS and PI3K/AKT signaling contribute to the proliferation of HASMCs. Numerous studies have demonstrated ISL could inhibit ROS generation and serve as an inhibitor of PI3K/AKT signaling. Therefore, we investigated whether treatment with ISL would have influences on ROS production and PI3K/AKT signaling in HASMCs. We found that ISL could significantly inhibit ROS production and the expressions of molecules in PI3K/AKT signaling. And H2O2 administration partially abolished the inhibitory effect of ISL on HASMCs proliferation. We also found that H2O2 could reactivate PI3K/AKT signaling in ISL treated HASMCs.

These results suggest that the ROS-scavenging property of ISL plays a crucial role in inhibiting the proliferation of HASMCs. Our data also reveal that PI3K/AKT signaling is an effector of ROS in regulating HASMCs proliferation. Overall, this study provides additional evidence to support the view that ISL play roles in protecting cardiovascular disorders.

## Methods

### Cell culture and treatment

Primary HASMCs was purchased from YRgene (NC008, China) and preserved in our lab. The cells were maintained in Dulbecco’s modified Eagle’s medium (DMEM, Gibco, USA) supplemented with 10% fetal bovine serum (Gibco, USA) at 37 °C in an atmosphere of 5% CO_2_ and 95% air. ISL with a purity of 98% was purchased from Sigma Aldrich (Japan) and dissolved in DSMO. HASMCs were treated with ISL alone, H_2_O_2_ alone or ISL combined with H_2_O_2_ at designed concentrations and time points. Cells cultured in the medium containing DMSO were used as a control.

### Cell Counting Kit-8 assay

Cell viability was detected by a Cell Counting Kit-8 (CCK-8, beyotime biotechnology of Nanjing, China). Cultured HASMCs were passaged onto 96-well culture plates (Corning, USA) at a density of 1× 10^4^ cells/well. After being treated with ISL, H_2_O_2_ or ISL combined with H_2_O_2_, CCK-8 assay was performed in accordance with the manufacturer’s instructions. The absorbance was determined at 450 nm by microplate reader (Thermo fisher scientific, USA). Cell viability was calculated relative to untreated HASMCs.

### Brdu incorporation

HASMCs were planted onto 96-well plates (Corning, USA) at a density of 1 × 10 ^4^/well and incubated with various dose of ISL for 48 h. BrdU cell proliferation ELISA kit (Abcam, USA) was used. Briefly, cells were treated with BrdU solution for 12 h. Afer cell fixation, anti BrdU antibody was added in the plates for 1 h in room temperature. Then, cells were washed three times in PBS and incubated with secondary antibody for 30 min at room temperature. Finally, after incubation with TMB and stop solution, the absorbance was determined at 450 nm by microplate reader (Thermo fisher scientific, USA).

### Flow cytometry analysis of cell cycle distribution

The cultured HASMCs were seeded on six-well plates at a density of 2 × 10^5^/well. The cells were treated with 10 μM ISL for 48 h and untreated cells were set up as control. For cell cycle analysis, cells were collected and re-suspended in 70% ethanol and stored at 4 °C overnight for fixation. The cells were washed in PBS for twice and incubated with propidium iodide (50 mg/ml) (PI; invitrogen, USA) at 4 °C for 30 mins. The samples were analyzed by flow cytometry (Becton Dickinson, USA); For cell death assay, cells were collected and re-suspended in PBS and incubated with propidium iodide (50 mg/ml) (PI; invitrogen, USA) at room temperature for 30 mins. The samples were analyzed by flow cytometry (Becton Dickinson, USA).

### Western blot

HASMCs were collected and lysed in RIPA buffer (pH 7·4) containing 2-amino-2-hydroxymethyl-propane-1,3-diol (Tris)-HCl (50 mM), NaCl (150 mM), EDTA (1 mM), Triton-X (1%), sodium deoxycholate (0.5%) and SDS (0.1%).

The homogenates were centrifuged at 13000 r/min for 15 min at 4 °C and the supernatant was obtained. Proteins were fractured by electrophoresis on a 10% SDS-polyacrylamide gel and transferred on onto PVDF membrane (Millipore, USA). Membranes were soaked in 5% milk/PBST for blocking.

Then, membranes were incubated with antibodies to p27kip1 (Abcam, USA), CyclinE (CST, USA), CyclinD1 (CST, USA), PI3K (Abcam, USA), p-PI3K (Abcam, USA), AKT (Abcam, USA), p-AKT (Abcam, USA) overnight at 4 °C. The membrane washed three times in PBST and Hrp-conjucated secondary antibody goat anti-rabbit or goat anti-mouse (Abcam, USA) were added on the membranes for 1 h in room temperature. Protein bands were visualized by staining with enhanced chemiluminescence (ECL).

### Flow cytometry analysis of ROS

HASMCs were digested by trpsin and re-suspended in 1 ml 2% BSA (beyotime biotechnology of Nanjing; China)/PBST. The cells were stained with DCFDA (2′,7′-Dichlorofluorescin diacetate, Sigma Aldrich, Japan) in dark at 37 °C for 30 min. Then the cells were washed three times in PBS and subjected to flow cytometry analysis (Becton Dickinson, USA).

### Biochemical test of SOD activity

HASMCs were collected and homogenized in 10% salt solution. The homogenate was centrifuged at 13000 r/min at 4 °C for 10 min and the supernatant was collected for biochemical detection. Commercial kit (Nanjing Jiancheng Bioengineering Institute, China) was purchased for SOD activity determination. The procedures were conducted in accordance with the instructions of manufacturer.

### Data analysis

Datas are expressed as mean ± SD. Unpaired Sudent’s test was used for comparison between two groups. For more than two groups one-way ANOVA was used and after ANOVA, Bonferroni test was used. *P* < 0.05 is indicated as statistically significant.

## Abbreviations

HASMCs: Human arterial smooth muscle cells
ISL: Isoliquiritigenin
ROS: Reactive oxygen species
SOD: Superoxide dismutase
VSMCs: Vascular smooth muscle cells
